# Water–Food Nexus Assessment in Agriculture: A Systematic Review

**DOI:** 10.3390/ijerph18094983

**Published:** 2021-05-07

**Authors:** Evelyn Corona-López, Alma D. Román-Gutiérrez, Elena M. Otazo-Sánchez, Fabiola A. Guzmán-Ortiz, Otilio A. Acevedo-Sandoval

**Affiliations:** 1 Área Académica de Química, Universidad Autónoma del Estado de Hidalgo, Carretera Pachuca-Tulancingo Km. 4.5, Ciudad del Conocimiento, Col. Carboneras, Mineral de la Reforma 42184, Mexico; co297894@uaeh.edu.mx (E.C.-L.); acevedo@uaeh.edu.mx (O.A.A.-S.); 2 CONACYT—Universidad Autónoma del Estado de Hidalgo, Mineral de la Reforma 42183, Mexico; fabiola_guzman@uaeh.edu.mx

**Keywords:** crops, CAPRI, GFWS, LCA, SWAT, WEAP, WF Nexus

## Abstract

The Water–Food Nexus (WF) has been proposed to reach equitable, balanced, and sustainable access to water and food resources in the face of the growing population demand. Therefore, developing models to assess them has become more relevant. This work systematically reviews the literature on the tools used to evaluate water and food resources between 2002 and 2020. Furthermore, it reports a critical analysis of the software used to assess the WF Nexus quantitatively. The models analyzed were Life Cycle Assessment (LCA), Common Agricultural Policy Regional Impact (CAPRI), Global Food and Water System (GFWS), Soil and Water Assessment Tool (SWAT), Water Evaluation And Planning system (WEAP), and Soil Water Atmosphere Plant (SWAP). We deduced that the following are necessary in evaluating the WF Nexus: (1) the capacity to generate future scenarios, (2) a global application, and (3) the application in case studies. The present paper is the first review to provide an overview of the software applied to evaluate WF Nexus, including the advantages and disadvantages of the tools found. They can help build sustainability criteria when designing policies that reduce water and food security risks and promote efficient water and food use.

## 1. Introduction

Water and food resources worldwide are necessary to human life, whose demands are sharply rising in recent years due to the growing population [1,2,3]. The demand for these resources is estimated to increase by over 50% by 2050 compared to 2015 [4]. Water resources assessment has become one of the leading global focuses. It is fundamental to ensure food supply and reach a global sustainable development in the face of population growth and climate variability [5,6,7]. In this context, the WF Nexus arises from an approach that promotes natural resource management’s interconnection and the importance of guaranteeing universal rights to these resources [8]. Therefore, new methods are designed for scenario predictions such as adaptation and mitigation simulating water and food security proposals for good governance [9,10,11,12,13].

### 1.1. The Water–Energy–Food Nexus (WEF)

The WEF Nexus’ popularity dates back to 2011 when the relationship of global challenges was acknowledged [14]. During the last two decades, this concept has been used to strengthen the synergic integration of the sectors covering a nexus and sustainable water use [14,15], which merge with Sustainable Development Goals (SDG) [16,17].

After WEF Nexus was launched in Bonn [14], many papers arose about a deep insight on the concept, interactions, the sustainable use of resources. Table 1 gathers selected published reviews about the WEF Nexus concerning hydric and food security in its political and social dimension.

The WEF Nexus convenience has been controversial [28,29]. However, all authors agree on its usefulness as an approach for decision-making, policy creation, and integration of resources management [26,30]. Several government organizations have declared the need for establishing measures that can lead to SDG compliance and guarantee the necessary resources for the present and future generations.

### 1.2. The Water–Food Nexus (WF)

In recent years, the WF Nexus has become an essential issue for the scientific communities due to the future uncertainty regarding safe access to resources that are essential to life [31]. The interconnection between water and food resources has led to a growing impulse to change management approaches [26]. The WF Nexus attracts the attention to ensure those resources, and to do so; quantitative models should allow its evaluation.

**Figure 1:**
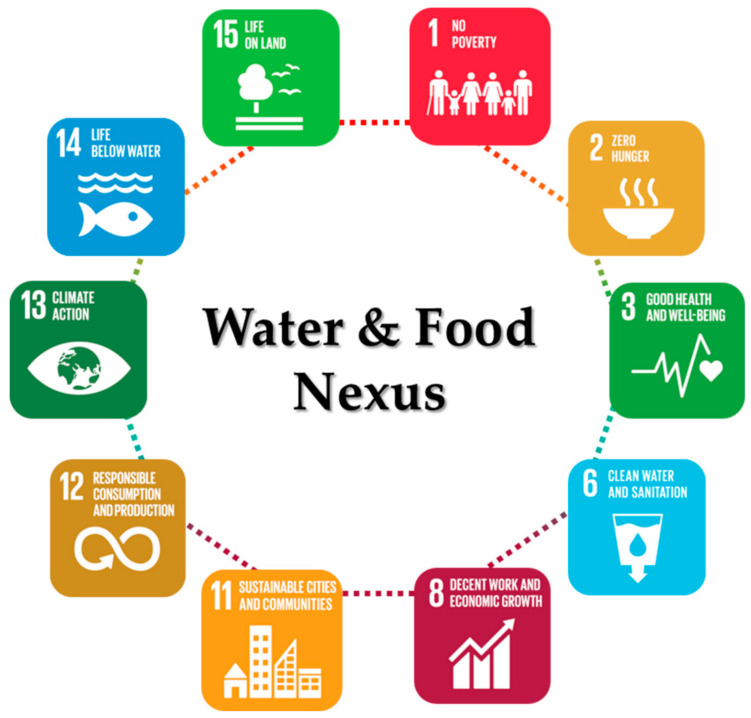
Interaction between the Water–Food Nexus and United Nations’ Sustainable Development Goals.

In the WF Nexus’s context, Figure 1 shows the relationship with 10 of the 17 United Nations’ Sustainable Development Goals [32]. According to this approach, the WF Nexus is key to the SDG’s fulfillment due to emerging challenges of hydric and food availability.

Some SDGs present an obvious connection with WFN (2, 3, 6, and 13), but the others have an indirect relation such as (1, 8, 11, 12, 14 and 15) because cities are resources’ consumers, agriculture creates jobs, unsustainable husbandry and agrochemicals affect life on land and underwater, and wellness is improved by agriculture goods.

The WF Nexus is a complex concept often used in the comprehensive study and management of global resource (water and food) systems [10,33]. The complexity of the nexus promoted several models to understand its scope better [22,34].

**Figure 2:**
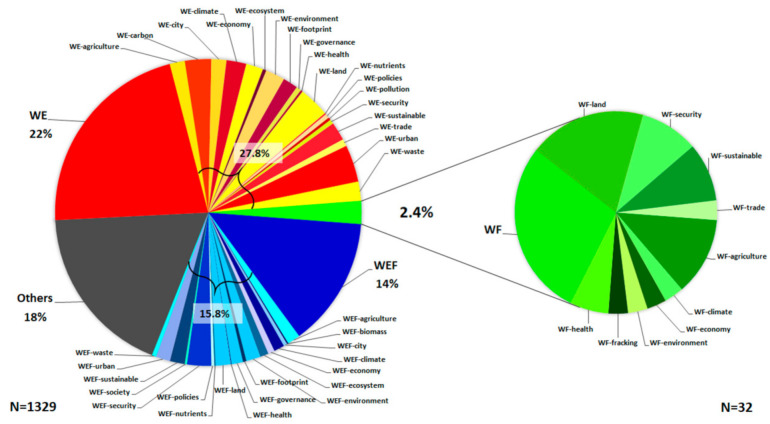
Published documents about Water–Energy–Food (WEF), Water–Energy (WE), Water–Food (WF), and others. SCOPUS analysis in 2002–2020.

Figure 2 shows the importance of the WF Nexus relevance, but few documents quantify it. As more research looks to sustainably satisfy human needs, regulating water and food resources is fundamental. It is essential to use a tool that promotes the WF Nexus evaluations and helps introduce new policies and resource management. The WF Nexus evaluation models are helpful to predict future scenarios in light of the shortage and demand of both resources.

Most of the published WF Nexus studies provide qualitative analyses, and few present a quantitative assessment, as observed in Figure 2. Then, analyzing those documents and their evaluation with a successful software could provide useful information for the scientific community, promoting this kind of research.

This work aims to provide a systematic review of the literature on the tools used to evaluate the WF Nexus that quantify these natural resources’ use to diagnose their sustainability degree. It will also allow for decision-making to create public policies. Then, we analyzed the models reported in the joint evaluation for water and food resources. We discuss the advantages and disadvantages of every software quantifying the WF Nexus. This article is the first review to deal with this topic.

The article is structured as follows: Section 1 introduces the WEF and WF Nexus. Section 2 provides the methodology and criteria to select the documents for this review. Section 3 presents the mathematical approaches used in its quantification on reported case studies, and discusses the advantages and disadvantages of used software. Finally, Section 4 briefs the conclusions.

## 2. Materials and Methods

A search on SCOPUS with the keywords’ water and Nexus yielded 1329 publications in 2002–2020.

Figure 2 shows the pie chart of published documents about the Water–Energy–Food (WEF) Water–Energy (WE), WF Nexus, interactions, and other variants. Research on WEF accounts for 29.8% (blue); WE gets the highest percentage, with 49.8% of papers (red), and WF reaches only 2.4% (green), which includes its interactions with land use, security, agriculture, climate, economy, and health. Pure WF accounts for 0.7%.

**Figure 3:**
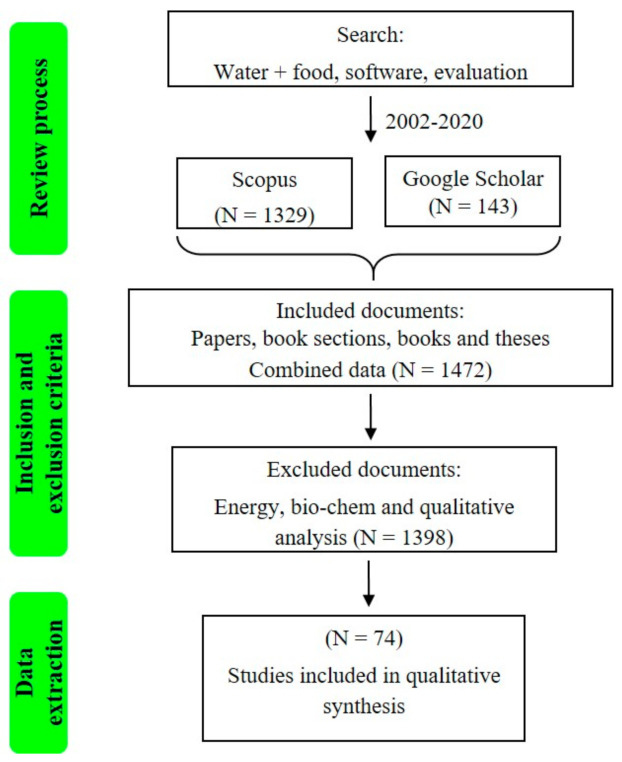
PRISMA according to [35] methodology criteria of the literature 2002–2020.

The documents’ selection follows the methodology scheme represented in Figure 3. Preferred Reporting Items for Systematic Reviews and Meta-Analyses–Extension for Scoping Reviews (PRISMA-ScR) methodology was used to evaluate the quality of the individual studies and score the body of evidence (BOE) [35]. Scientific engines were SCOPUS and Web of Science databases. The search terms were water, food, evaluation, Nexus, and quantification. Boolean phrases and words were ((KEY (water) AND KEY (Nexus) AND NOT TITLE (enamel or criminal or curing or extract* or therapy or dental or ceramic* or cement* or Westinghouse or urea or bio-chem*)) AND PUBYEAR > 2001), while the research date was 23 June 2020.

### 2.1. Review Process

The process consisted of a comprehensive search of keywords across databases, completed in three steps: identification, detection, and eligibility [35]. EndNote managed and organized the information by creating intelligent groups to identify the number of publications eligible for evaluation (see Figure 3).

### 2.2. Inclusion and Exclusion Criteria

The review includes published documents about hydric and food resources between 2002 and 2020. The inclusion criteria were (1) case studies of the WF Nexus; (2) the WF Nexus with any interaction; (3) use of tools to evaluate hydric, food, and agricultural resources; and (4) quantification of the Nexus; (5) theses, chapters, and books were admitted despite PRISMA recommendations. The exclusion criteria were energy, hydric resources, qualitative, and social issues.

### 2.3. Data Extraction

Full-text reading identified relevant papers about quantitative approaches and selected documents compiled into an information matrix that comprises the review’s body, including those about hydric and food resources assessment software (standardized and computational methods).

## 3. Results and Discussion

In this study, 74 articles met the eligibility criteria (see Figure 3) to review the WF Nexus tools described below.

### 3.1. Evaluation Models of the WF Nexus

The review compiles six reported models to evaluate water and food resources. Still, they were first created for purposes different from the evaluation of the WF Nexus. Table 2 shows the six leading tools found and their characteristics adapted for WF assessment.

As observed in Table 2, some models were designed around 50 years ago, and in time, they have been improved, and their current application is diverse. Six models are relevant, based on the review of the literature.

Life Cycle Assessment (LCA). This was developed in 1969 by Harry E. Teasley to evaluate a product or service’s environmental impact throughout its life cycle [42]. It represents an opportunity to improve product design, providing information to decision-makers in industry and government and non-governmental organizations (NGOs) [43]. Nevertheless, the LCA stage can be subjective, and its precision might be limited by the accessibility or quality of data. The standard ISO 14040/14044 LCA currently regulates LCA and involves economic, social, and environmental processes [44,45].

Water Evaluation and Planning system (WEAP). The model was designed in 1988 by Sieber and collaborators of the Stockholm Environment Institute (SEI-US) as software for integrating water resources, sustainability evaluation, and scenario design [37]. Its main feature is the integrated water resources analysis, modeling demand, and availability under different management and weather conditions [42,46]. WEAP is available by an online platform [47].

Soil & Water Assessment Tool (SWAT). This program was developed by Arnold and collaborators of the Agricultural Research Service-US Department of Agriculture (ARS-USDA) [38], based on a scaled model drainage basin to simulate the superficial and underground water amounts. It helps predict the environmental impact of land use, management practices, climate change, and the transportation of chemical products and nutrients in water and sediments [39]. Still, there are restrictions to simulate future water availability scenarios [46].

Common Agricultural Policy Regional Impact (CAPRI). This platform was designed by Wolfgang Britz, and the project is supervised by Thomas Heckelei, with the Bonn team’s contribution (U Bonn, EuroCARE), Thünen, SLU JRC-Sevilla, and JRC–Ispra [18]. It is a global partial equilibrium model for the agricultural sector, mainly focused on the European Union (EU). Used in evaluating the ex-ante impact of agricultural, environmental, and commercial policies, CAPRI was created to analyze agricultural scenarios [48]. In 2010, the CAPRI water module provided a scientific evaluation of agricultural water use in the EU and explored regional pressures on water resources [49].

Soil–Water–Atmosphere–Plant (SWAP). This program is the successor of SWATR, developed by Feddes and colleagues at the Wageningen University and Research (WUR) and published in 1978 [40]. It is a model designed to simulate flow and transport processes at a field scale during growing seasons and long-term time series [50]. The regional application within a geographical information system (GIS) environment demands additional features that are not currently included with the model.

The Global Food and Water System (GFWS). It is a platform created by the Food–Energy–Environment–Water (FE2W) Network, a group of 40 leading experts from universities, multilateral organizations, and NGOs [51]. The GFWS platform is designed to explore the relationship between the land surface and crop yield, water use, and fertilizers in the gap between production, demand, and water and food supply until the year 2050. Training and technical knowledge are needed using each of the programs above.

Comparing the models in Table 2, WEAP, SWAP, and SWAT provide the most outstanding advantage to evaluate water and food resources due to the ability to assess water’s influence on crops and the generation of scenarios. The connection of these models is the long-awaited software for the quantitative evaluation of the WF Nexus.

The literature review provided the case studies compiled in Table A1 Appendix A, in which the WF was calculated by any of the described models.

#### 3.1.1. Common Agricultural Policy Regionalized Impact (CAPRI)

CAPRI is a quantitative agricultural modeling system in the European Union whose primary goal is to analyze the farming policies’ economic impacts to safeguard food supplies at reasonable prices. The databases included in the model cover around 50 agricultural products for the EU [48]. Most of the studies that have used CAPRI are focused on evaluating climate change’s impact on agriculture, greenhouse gas (GHG) emission mitigation, and political-economic issues. As observed in Table A1, the use of CAPRI in evaluating hydric resources in crops is limited. The irrigation model included in CAPRI provides an evaluation of water used in agriculture and its pressures on the hydric resource in the EU [48,49]. According to the crop’s theoretical water demand, dry land, and crop irrigation and yield, the case study presented estimates the crop’s actual irrigation water use (CAWU) per irrigation area. Regional irrigation water use (IRWU) is calculated by adding those of each irrigated crop. This water model’s addition to CAPRI poses difficulties since it is not homogeneous and lacks precise EU data. Nevertheless, it represents a crucial step, and CAPRI is one of the main tools for the WF Nexus’s quantitative assessment.

#### 3.1.2. Global Food and Water System (GFWS)

GFWS is a platform available online to explore the relationships between crop yield, water use, food demand, and water supply, among others, for agricultural use with projections available up to 2050 [41]. The platform includes forecasts of population growth, calorie demand, diet changes, international commerce, and irrigation techniques. Data for the scenarios are available on the Organization for Economic Cooperation and Development (OECD), the United Nations (UN), and the Food and Agriculture Organization (FAO). The scenarios include 19 countries (Argentina, Australia, Bangladesh, Brazil, Canada, China, Egypt, France, India, Indonesia, Mexico, Pakistan, Poland, Russia, Thailand, Turkey, United States, Ukraine, and Vietnam) and their main crops: wheat, rice, corn, sorghum, barley, oat, and soybean [51].

GFWS forecasts the national agricultural water use (Wkj) in terms of the existing climate and using irrigation rates and land use areas, as shown in Equation (1):

W_kj_ = R_kj_ LI_kj_ × E_kj_ (1)

W_kj_ is the irrigation rate for crop k in country j, LI is the area of irrigated land for crop k in country j, and E is the efficiency rate of water use identified through the irrigation method for crop k in country j.

GFWS is an open access platform where the user can estimate the water supply so that agriculture can meet the national food requirements [52]. Until now, the use of GFWS is limited, as shown in Table A1; still, it is the basis to create indexes that allow reducing conflicts in the WF Nexus.

#### 3.1.3. Life Cycle Assessment (LCA)

The LCA analysis has been used to evaluate the environmental impacts attributable to all stages of the practical lifetime-use of products, services, or activities related [53]. Strategic management and decision-making models improve goods and services’ environmental performance. The ISO 14,040 standards the methodology based on the resource use and emissions associated with a production system and evaluates possible environmental impacts. In recent years, LCA’s environmental impact assessment in the production of several crops has increased [54,55]. LCA is suitable to endorse the optimal water management measures in food [56].

As shown in Table A1, LCA evaluates crops like corn, wheat, organic rice, tomato, cherry tomato, bell pepper, zucchini, melon, and soybean. The improved LCA methodology includes indicators as water use (WU) m3 year–1 and land use (LU) m2 year–1 [54]. This model is valuable within the WF Nexus context when used to assess water resources in agriculture.

Some studies reported the LCA’s performance in agriculture environmental impact assessment, mainly in Asia and Europe. The method to calculate water use impact, defined in ISO 14046, has been included [43]. This methodology provides for the water life cycle inventory considering water scarcity [55], evaluating water risks and potential impact on a crop’s life cycle. As a result, appropriate design of strategies and plans can minimize impact and provide information to decision-makers in the industry, NGOs, and government organizations.

LCA is also helpful for producers, consumers, and politics by adding reference values of water consumption in agriculture [57]. Nevertheless, there are no reports for scenario forecasting in water and food availability resources. LCA is more likely to be considered the WF Nexus assessment to evaluate the water use impact in a life cycle crop.

#### 3.1.4. Soil and Water Assessment Tool (SWAT)

This tool is used for river basins, predicts management practices’ convenience [58], hydrologic impact studies, even where data are limited [59]. It demands specific information on climate, soil properties, topography, vegetation, and soil management practices. It is a large-scale model used to simulate river basins, water quality, water balance, and crop yield, among others [60,61].

SWAT aims to predict agricultural practice management’s long-term impacts (crop rotation, planting date, harvesting, irrigation, fertilizers, and pesticide application times) [62].

Uniyal, Dietrich, Vu, Jha, and Arumí (2019) reported the application of SWAT to evaluate the water footprint of field-scale crop production. In 2019, Uniyal (2019) used SWAT simulating the irrigation needs in four river basins (Chile, Germany, India, and Vietnam) and predicted a significant amount of water to save [63]. On the other hand, some authors have demonstrated that SWAT is ideal in the simulation of hydrologic and agricultural processes [31,64].

In general, SWAT is a valuable program to manage hydric resources in agriculture. In response to the forecasts indicating an increase in food production and decreased hydric resources, SWAT focuses on assessing irrigation systems in agriculture. The aim is to ensure a high yield in crop production, improve water productivity, and sustainably distribute hydric resources. SWAT is a public domain model and valuable for assessing the WF Nexus.

#### 3.1.5. Soil Water Atmosphere Plant (SWAP)

SWAP is a hydrologic model to simulate vertical water flow processes in soil, solute transportation, and evaporation during the growing seasons for crops at a field scale [65,66]. Table A1 shows the SWAP-related case studies are based on the Richards equation [67], this shown in Equation (2):

∂θ/∂t = C(h) ∂h/∂t = ∂[K(h)(∂h/∂z + 1)]/∂z − S(h)(2)

C is the water capacity (∂θ/∂h) (cm^−1^), θ is volumetric water content (cm^3^), t is time (day), S is soil water extraction, K is hydraulic conductivity (cm d^−1^), h is soil water pressure head (cm), and z is the vertical coordinate (cm), taken positively upward.

SWAP was used to evaluate the water cycle under water deficit in a double-cropping system in Beijing, China. This study indicated that SWAP is suitable for simulating the water cycle and evaluating irrigation practices [65]. Further research used SWAP to simulate the optimal irrigation schedule and groundwater load across hydrologic years in a double-cropping system [68]. This model is also valuable for evaluating the optimal practices for crops on saline soils [65].

This review identifies the SWAP model as the best for managing and optimizing irrigation practices in crops. Then, it would be helpful in the evaluation of the WF Nexus by managing sustainable water and food allocation.

#### 3.1.6. Water Evaluation and Planning (WEAP)

The Water Evaluation and Planning (WEAP) software simulates the demand, supply, runoff, streamflow, water storage, and pollution discharge and creates water quality [69]. The system allows for model adaptation and variable definition according to the user’s information [26].

Table A1 shows case studies using WEAP to analyze water needs reduction, evapotranspiration analysis, and water availability evaluation in crops. It includes equations, functions, and variables for hydric resource planning and models for crop requirements and yields.

WEAP is an efficient software to improve irrigation techniques and evaluate their impact on hydric resources globally [70]. According to the WF Nexus, the most significant advantage is creating agricultural scenarios that allocate resources and make integrated management plans to balance supply and demand and guarantee sustainable development.

## 4. Conclusions

This paper presents a systematic review of the quantitative approach to assess the Water Food Nexus. From 1329 documents, only 2.4% are about the Water–Food Nexus evaluation in case studies, which evidences the need for further research.

The WF quantitative assessment in those papers was carried out by different approaches and software: LCA, CAPRI, GFWS, SWAT, SWAP, and WEAP, allowing calculation of hydric and/or food resources simultaneously.

According to the present review analysis, the Water–Food Nexus quantitative approach presents three principal characteristics: (1) facility to create future scenarios, (2) applied globally, and (3) useful for evaluating case studies.

LCA is based on the ISO 14040/14044 standard and meets two out of three desirable features to evaluate the Water–Food Nexus; still, it does not provide future projections regarding resource availability and accessibility. GFWS and CAPRI allow global future scenario simulation, but not in case studies. While GFWS provides a country database worldwide, CAPRI evaluates food economically and hydric balance is only affordable to Europe. Notably, SWAT is particularly useful in case studies, assessing the impact of the hydric resource in agriculture, but it is limited for scenario simulations. Finally, WEAP and SWAP allow the assessment of hydric resources in crop production from worldwide to local studies and the scenarios simulation about water allocation and availability in crops.

Because of the above, WEAP and SWAP are the most comprehensive models to evaluate the Water–Food Nexus and all its features, in the present. WEAP is recommended for scenario projections in sectors such as industry, agriculture; or regions from basins to urban settlements, and includes the cost–benefit calculation for food production. On the other hand, SWAP considers the water transportation system in crops, soil, and atmospheric characteristics, including water quality.

Despite the fact that there is no software dedicated to evaluate and rate the WF Nexus, WEAP and SWAP were adapted and afforded the best possibilities to do it.

This paper is the first review to provide an overview of the models used in the Water–Food Nexus quantitative assessment and the pros and cons of each software used in the literature.

### Future Directions for Nexus Research

The published documents evaluating the Water–Food Nexus are scarce; therefore, the Nexus analysis’s quantitative approach is demanding. Consequently, an indicator scale is convenient to rate the Nexus, giving sustainability criteria for policy-makers to minimize hydric and food security risks, promoting the efficient use of both resources. Water and Food are evaluated separately, and no software does it simultaneously. Furthermore, the Nexus does not have specific indices to rank the sustainability or scale-based classification. Consequently, an index scale is convenient to rate the Nexus, giving sustainability criteria for policies to minimize hydric and food security risks, promoting both resources’ efficient use.

## Figures and Tables

**Table 1:** Some relevant reviews about the Water–Energy–Food Nexus (WEF).

Focus	Description	Reference
Sustainability	Strengths to develop “environmental livelihood security”.	[18]
	Transdisciplinary research, public politics, and strategies for environmental management.	[17]
	Challenges for integrating and optimizing the nexus components. Four case studies were analyzed.	[19]
Current state	WEF Nexus in regions. Keywords and research for stakeholders’ understanding.	[20]
	Initiatives frame with involved actors. Challenge to achieve disciplinarity and boundary-crossing endorsed by the 2030 Agenda.	[21]
	State-of-the-art review on the concepts, research questions, and methodologies	[22]
	WEF Nexus analytical methods for knowledge-based approaches and promotion for further approaches.	[23]
	How the nexus approach has academically and geographically expanded	[24]
Social, political, and economic	The emerging literature on the WEF Nexus in the policy context	[25]
	Modeling tools to integrate policies.	[26]
	A modeling platform for the efficiency assessment of technologies, policies, and resources management planning.	[15]
	Circular economy approach for understanding the WEF Nexus interdependencies.	[27]

**Table 2:** Background of models adapted in case studies to assess the Water–Food Nexus.

Tools	Developer	Application	Advantages	Limitations	Reference
Life Cycle Assessment (LCA)	Harry E. Teasley, 1969	Environmental impacts	Identify hotspots	Interpretation can be subjective	[36]
Water Evaluation And Planning system (WEAP)	Jack Sieber, SEI 1988	Assessment of water resources	Dynamic simulation of scenarios	Does not separate ground and surface water demands	[37]
Soil & Water Assessment Tool (SWAT)	Jeff Arnold, USDA 1991	Assess of water resources and hydrological simulation	Simulates the transport of nutrients in water and sediment	Restriction for simulate future scenarios of water availability	[38,39]
Common Agricultural Policy Regional Impact Analysis (CAPRI)	ILR, UE 1997	Impact of agricultural policies	Analysis of agricultural scenarios	Global average coverage	[18]
Soil, Water, Atmosphere, and Plant (SWAP)	Reinder Feddes, WUR 1978	Use of water in crops	Simulates water transport in interaction with vegetation	It does not have a graphical user interface	[40]
Global Food and Water System (GFWS)	Quentin Grafton, 2014	Simulation platform	Simulation platform	Simulation platform	[41]

## Data Availability

The data presented in this study are available on request from the corresponding author.

## Appendix A

Table A1 compiles the models used for the evaluation of water and food resources. The models’ selection demands an understanding of the main goal, application area, time analysis, space scales, complexity, and information data to feed them.

**Table A1:** Assessment programs for the Water–Food Nexus: case studies.

Software	Objective	Indicator	Crop	Reported Value	Country	Reference
Common Agricultural Policy Regionalized Impact (CAPRI)	Agricultural and water modeling	IRWU_ri_ = Σ_wact_ CAWU_ri,wact_ × LEVL_ri,wact_^1^	50 agricultural products	IRWU: 3633.93 E6 m^3^	United States	[48,49]
Global Food and Water System (GFWS)	Assessment of food and water availability	W_kj_ = R_kj_ × Ll_kj_ × E_j_ ^2^	wheat, rice, corn, sorghum, barley, oats, and soybeans	water consumed by crop: 4 × 10^−7^ m^3^/ha	20 countries	[51]
Life Cycle Assessment (LCA)	Water consumption	Crop-rotation	wheat grain maize grain	437.5 m^3^/t of grain 232.2 m^3^/t of grain	China	[54]
	Water consumption	Water scarcity footprint (rice) = Irrigation water use (rice) × WSI ^3^	paddy rice	1.24 m^3^ H_2_O_eq_/kg paddy rice	Thailand	[55]
	Water consumption	Environmental performance	tomatoes cherry tomatoes peppers zucchinis melons	147.8 m^3^ 111.8 m^3^ 172.4 m^3^ 88.9 m^3^ 77.7 m^3^	Italy	[57]
	Irrigated with groundwater and reclaimed water	GW: irrigated crops with groundwater and RW: reclaimed water	corn soybean wheat	GW:0.44, RW: 0.37 GW: 0.39, RW: 0.37 GW: 0.64, RW: 0.56	China	[71]
Soil and Water Assessment Tool (SWAT)	Water footprint	SW_t_ = SW_0_ + Σ (R_day_ − Q_surf_ − E_a_ − W_seep_ − Q_gw_) ^4^	wheat corn sunflower	1.036 m^3^/kg 0.774 m^3^/kg 1.510 m^3^/kg	China	[39]
	Water requirement	wstr = 1 − E_t,act_/E_t_ = 1 − W_acualup_/E_t_ ^5^	rice, potato, sugar beet, winter wheat, oats	Deficit irrigation (25–48%) Reduced yield (0–3.3%)	India, Germany, Chile, and Vietnam	[63]
	Evaluation of change in irrigation systems	CPD = ΣY_i_ × A_i_/ΣV_i_ × A_i_ ^6^	wheat, apple, potato, tomato, sugar beet, alfalfa, and barley	Base scenario CPDiᴘ: 0.87 kg/m^3^ CPDᴇᴛ: 1.78 kg/m^3^ Increasing irrigation CPDiᴘ: 1.25 kg/m^3^ CPDᴇᴛ: 2.06 kg/m^3^	Irán	[31]
	Basin-scale hydrological model	WYSF: lower harvest index HVSTI: harvest index for optimal growing conditions	grain sorghum sweet sorghum	HVSTI: 0.45 WYSF: 0.25 HVSTI: 1.0 WYSF: 1.0	EE.UU.	[64]
Soil–Water-Atmosphere-Plant (SWAP)	Water cycle assessment	∂θ/∂t = ∂/∂z [K(h) (∂h/∂z + 1)] − S(h) ^7^	corn and wheat	saving water: 190 mm/yr groundwater recharge: 16.1 mm/yr	China	[72]
	Land management and water use	S_p_(z) = L_root_ (z)/∫^0-^_Droot_ L_root_(z)dz ^8^	grassland and corn		Holland	[73]
	Irrigation scheduling and groundwater recharge	C(h) ∂h/∂t = ∂/∂z [K(h)(∂h/∂z + 1)] − S_a_(z) ^9^	corn and wheat	optimal irrigation of 130, 260 y 390 mm in hydrological years of 25%, 50%, and 75%, respectively	China	[68]
	Performance and water use evaluation	∂θ/∂t = ∂/∂z [K(h) (∂h/∂z + 1)] − S(h) ^7^	corn	irrigation: 229 mm–460 mm	China	[65]
Water Evaluation And Planning (WEAP)	Reduction of crop water requirements	ADW: Alternate Wetting and Drying Ten years’ average	rice	54.88 Mm^3^	Philippines	[70]
	Evapotranspiration analysis	1981–2008 and 2011–2014	corn rice wheat	114mm 164mm 38mm	California	[74]
	Assessment of water availability	Average annual irrigation demand for water	yams, cassava, cocoa, rice, maize and tomatoes.	~690–748 Mm^3^/year	Africa	[42]

^1^ IRWU: Irrigation Regional Water Use. CAWU: Crop actual irrigation water use. LEVL: hectares cropped. ri: regions with irrigation. wact: total irrigated area. ^2^ W_kj_: agricultural water for crop k in country j. R: irrigation rate for crop k in country j. LI: area of irrigated land for crop k in country j. E: water use efficiency in country j. ^3^ WSI: Water Stress Index. ^4^ SW_t_: final soil water content in time t. SW_0_: initial soil water content. R_day_: the amount of precipitation on a day i. Q_surf_: the amount of surface runoff on a day i. E_a_ amount of actual evapotranspiration on a day i. W_seep_: the amount of percolation and bypass flow exiting the bottom of the soil profile in one day i. Q_gw_: the amount of return flow on a day i. ^5^ wstr: water stress. E_t_: maximum plant transpiration E_t,act_: the actual amount of transpiration. W_actualup_: total plant water uptake. ^6^ CPD: Crop index per drop. i: crop number. n: number of cultivated crops. Y_i_: yield of crop i. A_i_: area of crop i. V_i_: consumed water volume of crop i. ^7^ θ: soil water content in time t. dz: the vertical coordinate taken as positive upwards (cm). K(h): is the hydraulic conductivity specified by Van Genuchten–Mualem model (cm/d). S(h): represents the water extraction by plant roots (1/d). ^8^ S_p_(z): Stresses due to dry or wet conditions and/or high salinity concentrations may reduce. L_root_: the root length density (cm^−2^). D_root_: the root layer thickness (cm). ^9^ C(h): differential soil water capacity in soil water pressure head h. t: time. Z: vertical coordinate. K: hydraulic conductivity. S_a_: soil water extraction rate by plant roots
